# Tissue Specific Differentiation of Human Chondrocytes Depends on Cell Microenvironment and Serum Selection

**DOI:** 10.3390/cells8080934

**Published:** 2019-08-19

**Authors:** Annemarie Ecke, Anne-Helen Lutter, Jenny Scholka, Anna Hansch, Roland Becker, Ursula Anderer

**Affiliations:** 1 Department of Cell Biology and Tissue Engineering, Institute of Biotechnology, Brandenburg University of Technology Cottbus-Senftenberg, Universitaetsplatz 1, 01968 Senftenberg, Germany; 2 Center for Orthopaedics and Trauma Surgery, Brandenburg Hospital, Brandenburg Medical School Theodor Fontane, 14770 Brandenburg/Havel, Germany

**Keywords:** chondrocytes, cartilage, serum, differentiation, 3D culture techniques, fetal bovine serum (FBS), human serum

## Abstract

Therapeutic options to cure osteoarthritis (OA) are not yet available, although cell-based therapies for the treatment of traumatic defects of cartilage have already been developed using, e.g., articular chondrocytes. In order to adapt cell-based therapies to treat OA, appropriate cell culture conditions are necessary. Chondrocytes require a 3-dimensional (3D) environment for redifferentiation after 2-dimensional (2D) expansion. Fetal bovine serum (FBS) is commonly used as a medium supplement, although the usage of a xenogeneic serum could mask the intrinsic behavior of human cells in vitro. The aim of this study was to compare human articular chondrocytes cultivated as monolayers (2D) and the development of microtissues (3D) in the presence of FBS with those cultivated with human serum (HS). Evaluation of the expression of various markers via immunocytochemistry on monolayer cells revealed a higher dedifferentiation degree of chondrocytes cultivated with HS. Scaffold-free microtissues were generated using the agar overlay technique, and their differentiation level was evaluated via histochemistry and immunohistochemistry. Microtissues cultivated in the medium with FBS showed a higher redifferentiation level. This was evidenced by bigger microtissues and a more cartilage-like composition of the matrix with not any/less positivity for cartilage-specific markers in HS versus moderate-to-high positivity in FBS-cultured microtissues. The present study showed that the differentiation degree of chondrocytes depends both on the microenvironment of the cells and the serum type with FBS achieving the best results. However, HS should be preferred for the engineering of cartilage-like microtissues, as it rather enables a "human-based" situation in vitro. Hence, cultivation conditions might be further optimized to gain an even more adequate and donor-independent redifferentiation of chondrocytes in microtissues, e.g., designing a suitable chemically-defined serum supplement.

## 1. Introduction

Osteoarthritis (OA) is the most common degenerative joint disease with an estimated 250 million affected individuals worldwide [1]. The mean age for the diagnosis of osteoarthritis of the knee is 55 years with a mean expectation of 10 to 15 years before total knee surgery [2]. It is the leading cause of chronic pain and osteoarthritis-attributable mobility limitations, especially in the elderly. The Osteoarthritis Research Society International (OARSI) defines OA as a disease characterized by “cell stress and extracellular matrix degradation initiated by micro- and macro-injuries that activates maladaptive repair responses, including proinflammatory pathways of innate immunity” [3]. The functional disorder in the knee joint leads to an abnormal metabolism with structural changes in the entire joint resulting in cartilage degradation accompanied by enhanced bone remodeling, an inflammation of the joint, and eventually the loss of normal joint function leading to severe OA [3]. In addition to age-related changes in the joint structures [1,4], strong risk factors for knee OA include obesity, female sex, and a previous knee injury [5,6]. Moderate risk factors are a malposition of the knee, which leads to biomechanical stress, increasing the risk of OA in the more load-bearing region of the joint [7], leg inequality with a higher risk for a shorter limb [8], and muscle weakness of the knee extensor [9]. The resulting limitations in mobility are accompanied by swelling and pain in the knee and can lead to reduced life quality and productivity with an increased use of medical services, which ultimately results in increased morbidity [10]. 

Chondrocytes are the only cell population within articular cartilage, responsible for the anabolic and catabolic processes necessary to maintain cartilage health and function. The adult cartilage is avascular and contains a limited number of chondrocytes (less than 5%) [11], and the self-healing capacity of cartilage is very low. In addition to mechanical degradation during osteoarthritis, the chondrocytes change their normal behavior and begin to contribute to matrix degradation and abnormal matrix production, which is characterized by abnormal proliferation and/or apoptosis [12]. 

Conventional therapy options for OA are limited. In addition to weight loss, biomechanical interventions, and gentle exercises of the lower extremities [13,14], OA therapy consists primarily of pain management or anti-inflammatory medication [15], eventually resulting in total knee joint replacement with a limited lifespan of the prostheses. Therefore, alternative therapies, such as autologous cell-based methods, are coming more into focus. These therapies are transferred to the clinic to regenerate traumatic defects with good results [16,17]. The therapeutic benefit of transplanting monolayer (2-dimensional (2D)) cells into damaged tissue is limited due to the reduced viability of the cells exposed to the complex conditions in the microenvironment of the defect and the lack of cell-cell and cell-matrix interactions [18]. Cells in a 3-dimensional (3D) arrangement develop physiological interactions within an extracellular matrix (ECM) that reflect the situation in the native tissue [19]. These cells change, e.g., their expression of cell surface markers, making them less likely to trigger an immune response [18]. Chondrocytes isolated from cartilage tissue dedifferentiate in a 2D microenvironment and lose cartilage-specific ECM markers. On the other hand, increased proliferation in monolayer cultures enables cell production in larger quantities. Subsequent redifferentiation in a 3D microenvironment leads to the formation of round cell aggregates, the spheroids [20,21]. The spheroid technology is based on the use of autologous healthy cartilage cells of patients to produce dense scaffold-free microtissues in vitro [22], which are then implanted into the damaged cartilage area [23].

In order to make this method accessible to patients with osteoarthritis, it is necessary to analyze the quality (vitality, differentiation potential) and suitability to repair OA defects of chondrocytes isolated from condyles of patients with osteoarthritis. Therefore, the aim of this study was to isolate chondrocytes from human OA tissue, cultivate the cells in 2D to enlarge the total cell number, and redifferentiate the cartilage cells after this proliferation phase using a scaffold-free 3D microtissue culture technique. Various factors influence the proliferation and differentiation of chondrocytes in vitro, e.g., growth factors, vitamins, or hormones [20]. However, the special focus of this study was to evaluate the influence of different serum types on proliferation and differentiation of human chondrocytes in 2D and 3D culture.

The use of fetal bovine serum (FBS) as a growth factor supplement in media remains the current standard [24]. However, various studies have discussed the controversial use of FBS in cell culture technologies [24,25,26]. Major problems figure in the manufacturing process with unnecessary suffering of the unborn calf, the batch-to-batch variation in combination with an unknown serum composition, and the possibility of virus contamination [27]. Our aim was to investigate the replacement of FBS with human serum (HS) in the procedure of proliferation and differentiation of human chondrocytes in 2D and 3D culture using the agar-overlay technique to generate microtissues. 

## 2. Materials and Methods

### 2.1. Isolation of Human Chondrocytes and Cultivation as a Monolayer (2D)

Human articular cartilage was obtained from femoral condyles of patients undergoing knee replacement surgery. All patients gave their informed consent. The study was conducted in accordance with the Declaration of Helsinki, and the protocol was approved by the Ethics Committee of the Brandenburg University of Technology Cottbus-Senftenberg (EK2017-8, date of approval: 10/27/2017). A representative piece of the cartilage samples was cut out, frozen, and stored in liquid nitrogen as reference. Chondrocytes were isolated as previously described [28]. Briefly, tissue disintegration was done by mechanical mincing with a scalpel, followed by enzymatic treatment using collagenase (350 U/mL in DMEM:Ham’s F12 (1:1), Biowest, Nuaillé, France; 300 rpm interval mixing for 20 h at 37 °C). The isolated chondrocytes were centrifuged at 300× *g* for 5 min and the supernatant was removed. The cell pellet was resuspended in the basal medium containing DMEM:Ham´s F12 (1:1), supplemented with 4 mM l-glutamine (Biowest) and 10% human serum (German Red Cross, Cottbus, Germany). Cells were plated and expanded in the monolayer culture (2D) at 37 °C and 5% CO_2_ and detached for subcultures using 0.05% trypsin/0.02% EDTA (Biowest). Chondrocytes from passage 2 (P2) were characterized by indirect immunocytochemistry and used for the generation of microtissues. Cartilage samples from three donors were included in this study (Table 1). 

### 2.2. Generation of Microtissues (3D) and Sample Preparation for Analyses

**Figure 1:**
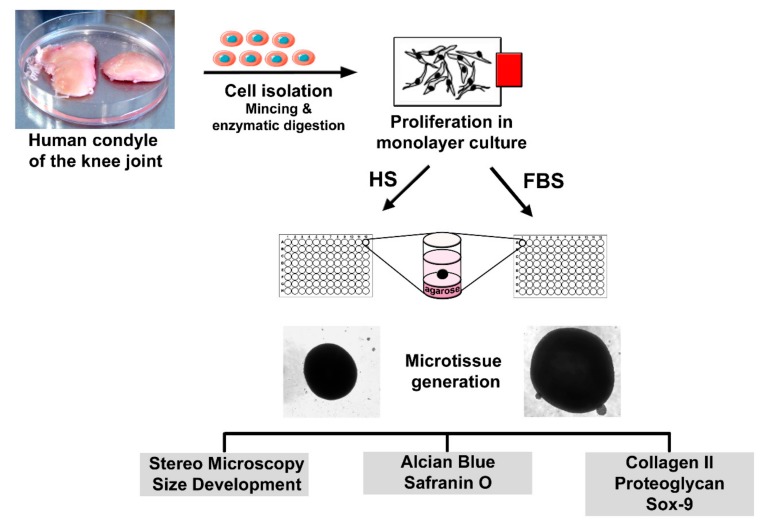
Experimental setup for the generation and analysis of scaffold-free microtissues derived from primary human articular chondrocytes.

Four days prior to microtissue initiation, cells were separated into two groups: One group of 2D cells was further cultivated in the basal medium with 10% HS, whereas the other group received a change in serum type. These cells were then cultivated in the basal medium containing 10% fetal bovine serum (Biowest). To analyze the differentiation degree of the cells prior to microtissue formation, P2 chondrocytes of both conditions were seeded onto glass slides and incubated at 37 °C and 5% CO_2_. After cultivation, the slides were washed with phosphate-buffered saline (PBS) and further processed for analysis of cartilaginous and non-cartilaginous markers. Scaffold-free microtissues were generated using the agar overlay technique as previously described [29]. Briefly, P2 chondrocytes were suspended in the differentiation medium containing DMEM:Ham’s F12 (1:1), 4 mM l-glutamine, ITS (Sigma-Aldrich, Munich, Germany) and 1% serum and seeded in agarose-coated 96-well plates at a density of 3 × 10^5^ cells/well. Medium changes were performed every other day. After 4 to 8 weeks of cultivation, microtissues were harvested, embedded in the Tissue Tek frozen section medium (Weckert Labortechnik, Kitzingen, Germany), and sectioned using a cryomicrotom (Microm GmbH, Walldorf, Germany) in 9 µm slices. The sections were allowed to air dry before being used for further analysis. A simplified overview of the cell isolation, microtissue formation, and sample analyses is shown in Figure 1.

### 2.3. Immunochemical and Histological Analysis

Indirect immunochemical analyses were carried out in monolayer-cultured chondrocytes and microtissues to detect the expression of collagen type I and II, proteoglycans (PG), Sox9, and Ki67. Sections from human cartilage served as control. The samples were fixed in 4% formaldehyde solution (AppliChem, Darmstadt, Germany) at 4 °C for 10 min, followed by 10 min at −20 °C in methanol/acetone (1:1; VWR, Darmstadt, Germany). For the detection of Ki67, samples were fixed for 5 min in methanol at −20 °C, followed by 2 min at −20 °C in acetone. The slides were allowed to air dry and were then rinsed with PBS. To visualize collagens in the tissues, the samples were incubated for 30 min at 37 °C with hyaluronidase (2 mg/mL; AppliChem) and rinsed with PBS. All samples were incubated with 2% normal goat serum (Dianova, Hamburg, Germany) in PBS/0.1% bovine serum albumin (BSA; Roth, Karlsruhe, Germany) for 20 min at room temperature (RT). Primary antibodies were diluted in PBS/0.1% BSA as follows: Anti-collagen type I was diluted 1:800 (MP Biomedicals, OH, USA), anti-collagen type II 1:600 (MP Biomedicals), anti-PG 1:100 (Sigma-Aldrich), and anti-Ki67 1:50 (DakoCytomation, Glostrup, Denmark). For the detection of Sox9, fixation was carried out for 15 min at RT in 4% formaldehyde solution, followed by rinsing of the slides with PBS. After incubation with 5% goat serum in PBS/0.3% Triton X-100 for 1 h at RT, the primary antibody anti-Sox9 (Merck, Darmstadt, Germany) was added, diluted 1:500 in PBS/1% BSA, and supplemented with 0.3% Triton X-100. The slides were incubated with the primary antibodies at 4 °C overnight in a humidified chamber. After extensive washing with PBS, the samples were incubated with Cy3-conjugated goat anti-mouse (collagens type I and II, PG, and Ki67) or goat anti-rabbit (Sox9) secondary antibody (Dianova) for 1 h at RT. The secondary antibodies were diluted 1:600 in the respective dilution buffer of the primary antibody, including 0.1 µg/mL DAPI (Sigma-Aldrich) to stain the cell nuclei. The preparations were mounted in the fluorescent mounting medium (DakoCytomation) after rinsing with PBS. Histological analyses were performed on microtissue and cartilage sections with Safranin O-Fast Green (AppliChem) and Alcian Blue (Serva Electrophoresis, Heidelberg, Germany)-Nuclear fast red (Merck) to visualizes the glycosaminoglycans, following the standard procedure [30,31]. To evaluate the amount of collagen type I, type II, PG, and Ki67-expressing chondrocytes in the monolayer culture, a set of 9 images each was analyzed. The total cell number was determined by counting DAPI-stained cell nuclei using ImageJ software (version 1.52a; http:rsbweb.nih.govij), and the number of cells expressing the matrix molecules were counted manually. The evaluation was performed by determining the percentage of positive cells.

### 2.4. Visualization of Actin Structures in Chondrocytes

To visualize actin structures in monolayer-cultivated P2 chondrocytes, Alexa Flour 488-conjugated phalloidin (New England Biolabs, Whitby, ON, Canada) was used. Chondrocytes on glass slides were fixed for 10 min at RT in 4% formaldehyde solution and then rinsed with PBS. The slides were incubated with 0.1% Triton X-100/PBS for 20 min at RT and then rinsed with PBS. Afterwards, the slides were incubated with the blocking solution containing 0.05% Tween-20 (AppliChem) in PBS/1% BSA for 10 min at RT. Alexa Fluor 488-conjugated phalloidin was diluted 1:300 in the blocking solution containing 0.1 µg/mL DAPI. The slides were incubated for 1 h at RT. After extensive washing with PBS, the preparations were mounted in the fluorescent mounting medium.

### 2.5. Viability Analysis of Cells in Microtissues

After 4 weeks, the medium was removed from the wells and the microtissues were rinsed with PBS. After incubation for 5 min with 60 µM propidium iodide (Sigma-Aldrich) solution, the microtissues were thoroughly washed with PBS and fixed for 1 h at RT in 4% formaldehyde solution. The tissues were embedded in the Tissue Tek frozen section medium and sectioned using a cryomicrotom. To capture cells from all layers, sections through the center of the ball-shaped microtissues were transferred on glass slides and allowed to air dry. After rehydration in PBS, the sections were incubated with DAPI (0.1 µg/mL) and diluted in PBS for 30 min at RT. The sections were rinsed with PBS, mounted with the fluorescent mounting medium, and then analyzed.

### 2.6. Microscopic Analysis

Morphology of living cells and microtissues was observed using phase contrast microscopy (CKX41; Olympus, Hamburg, Germany) and documented with a DP71 camera (Olympus). Images and the diameter of microtissues were evaluated using Cell^D^-Imaging software (Soft Imaging Systems, Muenster, Germany). Furthermore, living microtissues were analyzed via reflected light microscopy (SZX10 stereo microscope, Olympus; DP71 camera and Cell^D^-Imaging software) to document the surface of the tissues. Fluorescence imaging was performed with a IX81 fluorescence microscope system (Olympus) with a xenon burner (MT20, Olympus). Images were taken with a monochrome camera (Retiga 6000; QImaging, Surrey, BC, Canada) and subsequently colored using cellSens Dimension software (Olympus). Histological preparations were documented with a BX41 microscope (Olympus) equipped with a ColorView I camera (Olympus).

### 2.7. Statistical Analysis

The average size of the spheroids was determined by measuring the diameter of 10 spheroids. Cells positive for collagen type I, type II, proteoglycans, and Ki67 were evaluated by analyzing 9 individual images of each staining. Results are displayed as mean ± standard deviation (SD). A significance analysis of the size measurement was performed using two-way ANOVA (not repeated measures) followed by Sidak’s multiple comparisons test using GraphPad Prism version 6.07 for Windows (GraphPad Software, La Jolla, CA, USA).

## 3. Results

### 3.1. Chondrocytes in 2D: Reduction of Chondrogenic Markers Paralleled by Augmentation of Proliferation

**Figure 2:**
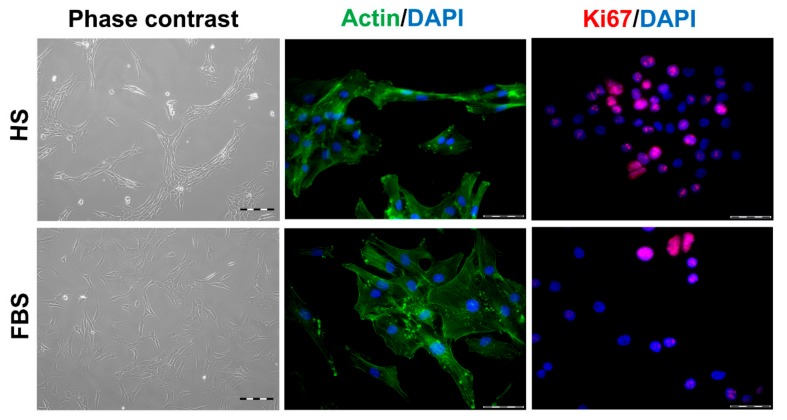
Morphology and proliferative activity of isolated chondrocytes of a representative donor. Passage 2 (P2) chondrocytes were cultivated with the medium containing either human serum (HS) or fetal bovine serum (FBS), and the morphology was documented via phase contrast microscopy (left). The cells were further analyzed via staining of actin structures using Alexa Fluor 488-conjugated phalloidin (green, middle). The proliferative activity was visualized via indirect immunocytochemical staining of Ki67 (red, right). Cell nuclei were stained with DAPI (blue). Scale bars: 200 µm (left) and 50 µm (middle and right).

**Figure 3:**
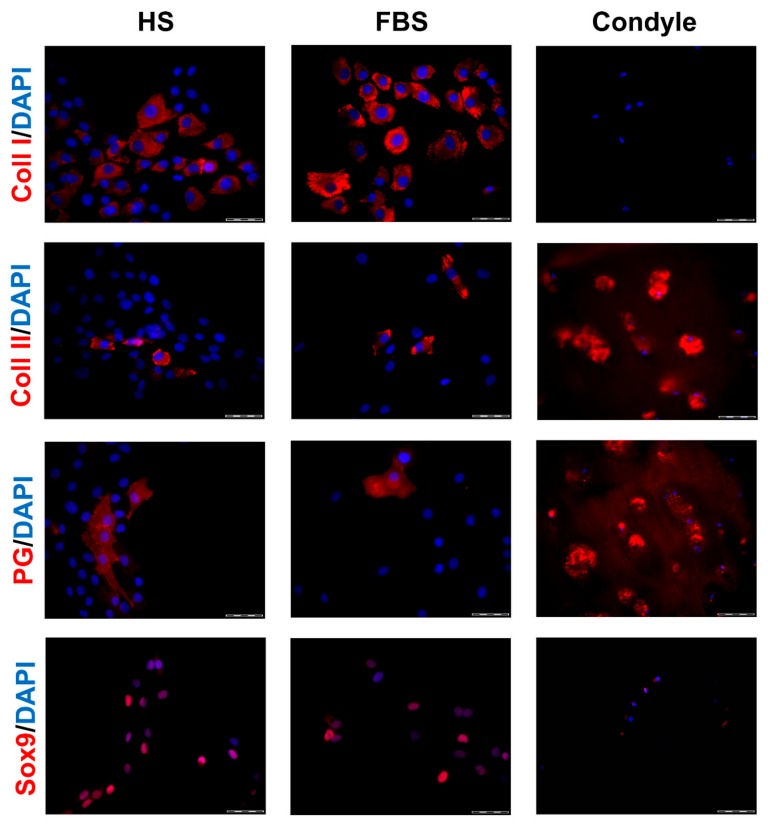
Expression of cartilage-specific markers in 2-dimensional (2D) chondrocytes of a representative donor. Immunocytochemical analyses on P2 chondrocytes cultivated with medium containing either HS or FBS. Sections from the condyle of the same donor is shown as reference. Red: Collagen type I (Coll I), collagen type II (Coll II), proteoglycans (PG), and Sox9, respectively. Blue: Cell nuclei. Scale bars: 50 μm.

**Figure 4:**
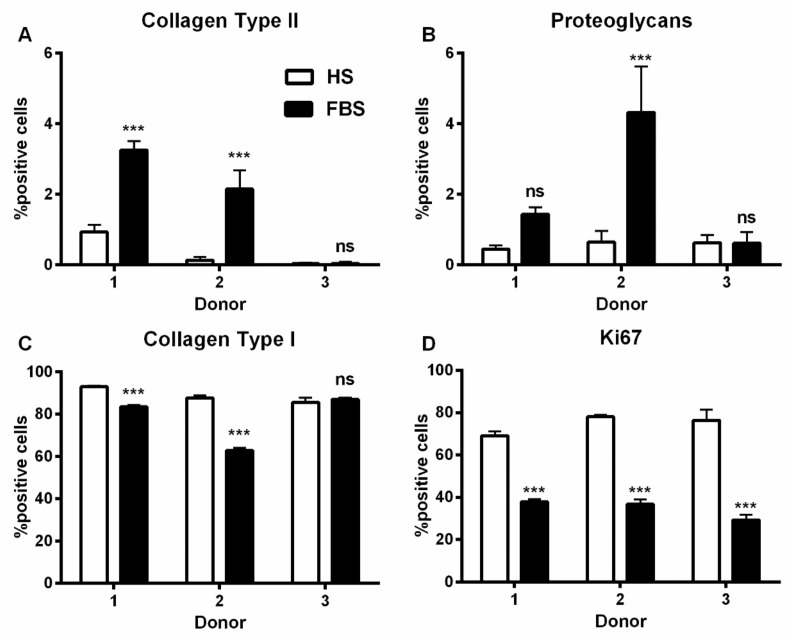
Percentage of chondrocytes expressing cartilage-specific matrix molecules. P2 chondrocytes were cultivated with the medium containing either HS or FBS. The expression of the cartilage-specific molecules collagen type II (**A**) and proteoglycans (**B**), the dedifferentiation marker collagen type I (**C**), and the proliferation marker Ki67 (**D**) were analyzed via indirect immunocytochemistry. (Not significant (ns), ***: *p* < 0.001).

During in vitro cultivation as monolayer (2D), chondrocytes of all donors showed hardly any chondrogenic features shortly after isolation and plating. The 2D cells presented an elongated fibroblast-like appearance (Figure 2, phase contrast), which was even more pronounced in higher passages. This cell stretching and spreading of 2D cells was also shown using the high affinity binding of phalloidin to the cytoskeleton element actin (Figure 2). Furthermore, the proliferation marker Ki67 was highly expressed in these monolayer cells (Figure 2). Results of the immunocytochemistry of the 2D cells compared to the original cartilage (condyle) showed a downregulation of the expression of cartilage-specific markers (collagen type II, proteoglycans) and an increase of the cartilage-unspecific protein collagen type I (Figure 3). The quantification of collagen type II and proteoglycans expressing cells reveals less than 5% positive cells for these cartilage markers (Figure 4A,B). Generally, the number of PG-expressing cells was higher compared to collagen type II. Collagen type I, on the other hand, was expressed by more than 60% of the cells (Figure 4C).

Although all chondrocytes dedifferentiated in 2D culture regardless of serum selection, distinct differences in morphology, proliferation, and expression of cartilage-specific molecules could be observed (Figure 1, Figure 2 and Figure 3). Chondrocytes cultivated in the medium containing FBS appeared to be bigger and more spread out compared to those cultivated in HS (Figure 1, middle row). Furthermore, cells in the medium with HS reached confluence faster than those with FBS during cultivation. This observation could be evidenced by significantly more Ki67-expressing cells and, thus, a higher proliferative activity for cells in HS (Figure 1 and Figure 4D). 

Generally, the amount of collagen type I and II as well as PG-expressing cells differed between the conditions (Figure 3). Chondrocytes cultivated in FBS showed more cartilage-specific ECM expressing cells with a partially higher intensity in staining than those in HS (Figure 3 and Figure 4). The expression of cartilage-unspecific collagen type I is significantly reduced in cells cultivated in FBS (Figure 4C). Furthermore, the magnitude of deviation between the sera varied from donor to donor. However, the cartilage-specific transcription factor Sox9, expressed in early chondrogenic determination, was expressed nearly ubiquitously in all 2D cell conditions in the nucleus and in the cytoplasm (Figure 3).

### 3.2. Chondrocytes in 3D: Differentiation Depends on Serum Type

**Figure 5:**
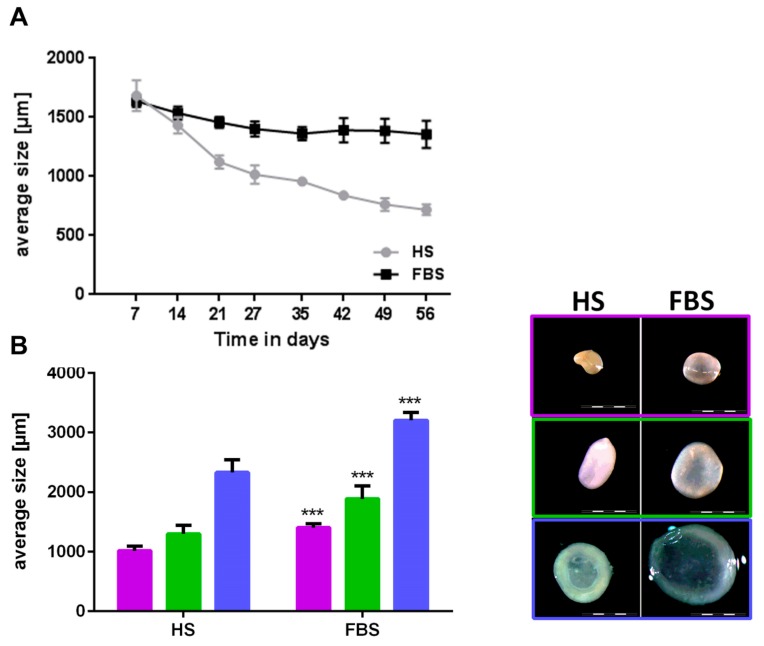
Comparison of size and macroscopic appearance of microtissues after cultivation in either HS or FBS. (**A**) Development of the size of microtissues from one representative donor over the course of 8 weeks. (**B**, left) Mean sizes of 10 microtissues of three donors after 4 weeks of cultivation. Each color represents a different donor. (**B**, right) Macroscopy of microtissues of the corresponding donors depicted in the graph. Scale bars: 2000 µm. Data are depicted as mean ± SD.

**Figure 6:**
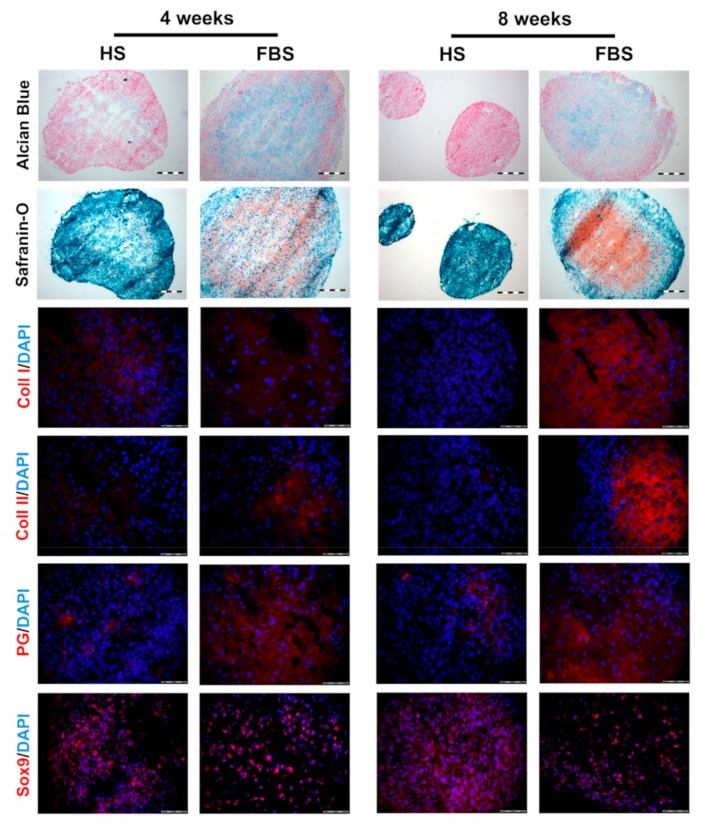
Comparison of the differentiation degree of microtissues from one representative donor after 4 and 8 weeks of cultivation using cryosections. (Upper rows) Histological analyses visualizing glycosaminoglycans (Alcian Blue: Blue, Safranin O: Red) and cell nuclei (Alcian Blue: Pink, Safranin O: Cyan). Scale bars: 200 µm. (Lower rows) Immunohistochemical analyses of cartilage-specific markers. Red: Collagen type I (Coll I), collagen type II (Coll II), proteoglycans (PG), and Sox9, respectively. Blue: Cell nuclei. Scale bars: 50 µm.

During the cultivation in a 3D environment, chondrocytes from all donors regained their cartilage-like features with distinct differences between both medium compositions. Chondrocytes regained a round cell shape indicated by little to no staining located closely around the cell nuclei of cytoskeleton elements such as vimentin (data not shown). Microtissues could be generated from all donors in both medium conditions and reduced their size over the course of 4 weeks (Figure 5A). Although microtissues of both medium compositions were similar in size at the first week after generation, microtissues cultivated in medium with FBS were significantly larger in size (diameter in FBS around 40% larger compared to HS) after 4 weeks (Figure 5B). Absolute values varied among the individual donors. While microtissues cultivated in medium containing HS further decreased their size over the course of 8 weeks, the size of those cultivated in FBS remained constant after 4 weeks (Figure 5A). This led to an even bigger size difference of 50 up to 80% larger microtissues in medium containing FBS compared to those in HS after 8 weeks. Reflected light microscopy revealed a smooth surface and a slight yellowish-to-white color of the microtissues after 4 weeks (Figure 5B) and 8 weeks (data not shown) in both medium compositions. The longer the spheroids were cultivated in HS and the smaller their size (diameter 1 mm and below), the more yellowish was their color, which indicated a less cartilage-like matrix within the microtissues cultivated in HS compared to those in FBS. Histological and immunohistological analyses confirmed this assumption (Figure 6). 

After 4 weeks cultivation, microtissues showed an increased collagen type II and proteoglycan expression when cultivated in the medium containing FBS compared to those in HS with no collagen type II and little PG expression. The expression of collagen type I was higher in HS than in FBS. Similar to chondrocytes cultivated as monolayers, Sox9 was ubiquitously expressed regardless of serum selection. The results of the histochemical analyses visualizing glycosaminoglycans was analogous to the immunohistochemical findings. Microtissues cultivated in FBS showed an almost complete staining of Alcian blue and Safranin O, whereas microtissues in HS showed a weaker signal in a smaller, restricted area in the middle of the tissues. However, in both cases, extracellular matrix molecules were predominantly found in areas where cell nuclei were widely scattered. After a further 4 weeks of cultivation, microtissues cultivated in FBS changed only slightly. Both collagen type I and II expression increased, whereas the collagen type II signal was stronger than that of collagen type I. The expression of proteoglycans and Sox9 were similar to 4 weeks. Microtissues cultivated in HS, on the other hand, showed a reduction in collagen type I and still no signal for collagen type II. Sox9 and proteoglycan expression were similar to 4 weeks, although the proteoglycans were even more restricted to one area of the tissue. Histological analyses of the microtissues showed the same trend: The Safranin O signal increased in microtissues cultivated in FBS, whereas the signal for both Alcian blue and Safranin O decreased in HS with only a very small positive area. The signal of Alcian blue decreased slightly in microtissues cultivated in FBS compared to 4 weeks. 

**Figure 7:**
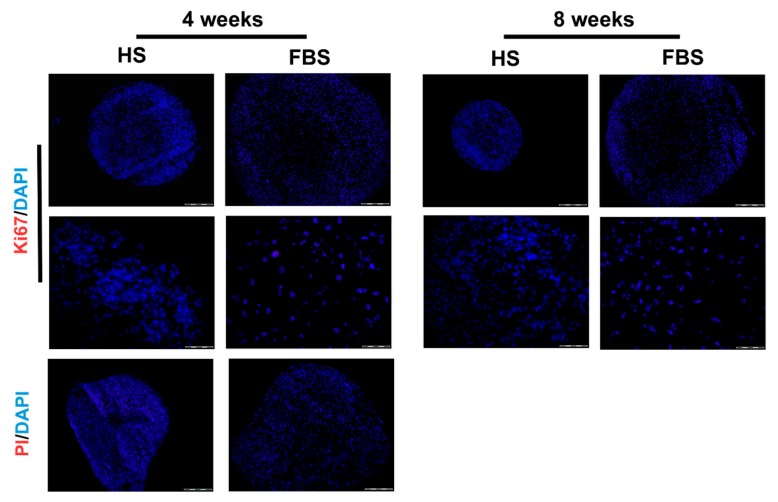
Analysis of cell proliferation and viability of microtissues from one representative donor cultivated for 4 weeks in the medium containing either HS or FBS. (Upper rows) Immunohistochemical analysis of the proliferation marker, Ki67. Red: Ki67. Blue: Cell nuclei. Scale bars upper row: 200 µm. Scale bars lower row: 50 µm. (Lower row) Viability staining using propidium iodide (PI). Red: Propidium iodide. Blue: Cell nuclei. Scale bars: 50 µm.

To prove that the size development of the microtissues was neither due to a cell loss nor due to cell proliferation, a viability staining test as well as immunohistochemistry for Ki67 was performed (Figure 7). Results revealed neither propidium iodide positive cells nor Ki67 positive cells in both serum conditions. This indicates that there are no necrotic regions within the microtissues and no proliferative cells that could have increased the cell amount.

## 4. Discussion

Conventional therapeutic options for OA are limited, consisting of pain management or anti-inflammatory medication [15]. Therefore, cell-based methods are a promising therapeutic alternative to pharmacological approaches. Technologies using cells in a tissue environment (microtissues or spheroids) have many advantages over the implantation of single cells or cell suspensions. The aggregation of cells in vitro leads to increased cell viability and improved differentiation capacity. The autologous spheroid-based implantation strategy can improve the prospect of repair in the case of injuries in the knee by placing chondrogenic microtissues with vital cells directly in the defect area [16,17]. In addition, there is an urgent need to identify therapies for OA patients, e.g., cell-based strategies. Here, it is necessary to improve the cell viability and pre-differentiation of cells in cartilage microtissues/spheroids to enhance implantation efficiency and advance cell-based therapies [18]. 

It is known that transferring chondrocytes from their natural environment into an artificial monolayer culture changes their typical expression profile. e.g., they express type I collagen instead of type II collagen, which is specific for hyaline cartilage [32,33]. This phenotypic shift was also observed in the cells of OA patients investigated in this study. Furthermore, the character of the phenotypic shift of chondrocytes in 2D culture is serum dependent. Cells cultured in HS revealed a higher reduction of the chondrogenic differentiation degree (Figure 3 and Figure 4) compared to cells in FBS. This high dedifferentiation correlates with an increased proliferation rate in cells cultured in the medium with HS. This observation goes hand in hand with several studies that have demonstrated increased proliferation of human chondrocytes cultured with HS [34,35,36]. An augmentation of proliferation in the medium plus HS was also observed, e.g., for human breast cancer cells in primary culture [37] and primary cultures of endothelial cancer cell lines [38]. For clinical application, it is of central importance to generate sufficient cell quantities for a therapy in as short a time as possible.

Monolayer cells of all donors formed tissue-like aggregates and showed a cartilage-like differentiation profile. This redifferentiation process was already well-developed after a 3D cultivation time of 4 weeks. The cultivation time for the differentiation of chondrocytes in 3D cultures in most studies is in the range of 2 to 4 weeks [39,40,41]. In order to check whether a significant extension of the cultivation time leads to a higher differentiation, the microtissues were cultivated for up to 8 weeks. However, an extension of the cultivation time up to 8 weeks showed only a slight increase of cartilage-specific markers (Figure 6). The cells in microtissues stopped to proliferate and were viable throughout the entire cultivation period (Figure 7). An extension of the cultivation time up to 8 weeks is possible with both HS and FBS. This allows long-term testing of, e.g., drug effects.

However, the differentiation of the microtissues was serum dependent. Serum additives in the medium are important for proliferation, differentiation, and maintenance of cellular activities. Serum provides essential compounds, such as hormones, vitamins, and adhesion factors, and is also involved in pH buffering or protease inhibition processes [26]. Microtissues cultured in FBS were bigger in size (Figure 5) and more highly differentiated (Figure 6) compared to HS. A higher expression of collagen type I (dedifferentiation marker) and type II (cartilage marker), as well as a higher expression of PG is opposed to an equally high expression of Sox9 in all culture conditions. The transcription factor Sox9 is early expressed in chondrocyte differentiation and is essential for the successive steps of chondrogenic differentiation [42]. In this respect, all cultivation conditions have the essential basis for chondrogenic differentiation, the expression of the transcription factor Sox9.

FBS as a traditional media supplement has several disadvantages, especially when working with freshly isolated human cells or developing cell-based therapies. In addition to the unknown composition and batch-to-batch variations, the probable contamination with endotoxins, viruses, and mycoplasmas has to be considered [24]. Furthermore, the species-specific composition of FBS could alter the properties of primary human cells in culture. The environment of primary human cells in culture should be as close as possible to the natural situation. This includes 3D cultivation as well as nutrient supply similar to the situation in the human body, which is the blood or the human serum, respectively.

Depending on the desired application, both HS and FBS could be an appropriate medium additive. Variations between different batches of HS and FBS can significantly change the results [36]. For large-scale research in experimental pharmacology, FBS would be a good supplement to produce microtissues generated from a chondrogenic cell line. Due to the large-scale production of FBS, a large quantity of a batch is commercially available. This ensures cost-effective long-term experiments with adequate reproducibility. In order to exclude species-specific influences on microtissues and prevent the possibility of prion or viral transmission and immune reaction against animal proteins [43], the use of HS would be recommended for work with human cells. 

In summary, it can be said that chondrocytes must be cultivated in a 3D environment in order to develop their tissue-specific differentiation. In a 2D culture, they reduce their typical characteristics and proliferate; a feature they do not exhibit in their normal environment in adult tissue. In addition to the spatial arrangement in a 3D pattern, the cell culture medium and selected additives are important parameters to obtain highly differentiated chondrocytes. Both serum supplements can be used to produce microtissues from isolated human chondrocytes, but the decision should take into account the advantages and disadvantages of adding HS or FBS. 

Further research approaches should focus on the identification of a chemically defined serum supplement. For preclinical in vitro studies with human chondrocytes, it may be also advisable to use autologous human serum to avoid batch-dependent influences and bring human cartilage tissue engineering nearer to a safe clinical application [44]. 

## Figures and Tables

**Table 1:** Characterization of donor samples.

Donor	Gender	Age	Macroscopic Appearance	Cell Yield	Cell Viability (%)
1	Female	57	Smooth, intact surface	5.3 × 10^6^	92.80
2	Male	49	Smooth, intact surface	3.34 × 10^6^	92.40
3	Female	73	Surface discontinuity, fibrillation visible	6.44 × 10^6^	91.75
